# Supplementary material for: Promiscuous specialists: Host specificity patterns among generalist louse flies
Source: PLoS One. 2021 May 27;16(5):e0247698. doi: 10.1371/journal.pone.0247698 (PMC8158981; doi:10.1371/journal.pone.0247698)
Supplement: S1 Fig — (DOCX) [file pone.0247698.s001.docx]

**S1 Fig.** Bird ringing sites in Finland, which contributed to the data collection.
