# Supplementary material for: Promiscuous specialists: Host specificity patterns among generalist louse flies
Source: PLoS One. 2021 May 27;16(5):e0247698. doi: 10.1371/journal.pone.0247698 (PMC8158981; doi:10.1371/journal.pone.0247698)
Supplement: S1 Table — The number investigated individuals per species (N) and number of parasitized hosts per species (Flies) are reported from all investigated birds (all) and only those birds which had been systematically sampled by ringers (syst). The prevalence of host species has been calculated based on systematic surveys (- = is no data) and values where less than 10 birds have been sampled are shown in brackets due to small sample sizes. (DOCX) [file pone.0247698.s002.docx]

**S1 Table**. List of 134 bird species which had been investigated by the bird ringers. The number investigated individuals per species (N) and number of parasitized hosts per species (Flies) are reported from all investigated birds (all) and only those birds which had been systematically sampled by ringers (syst). The prevalence of host species has been calculated based on systematic surveys (- = is no data) and values where less than 10 birds have been sampled are shown in brackets due to small sample sizes.

| Species | N all | Flies all | N syst | Flies syst | Prev |
| --- | --- | --- | --- | --- | --- |
| *Accipiter gentilis* | 2 | 2 | 2 | 2 | 1.00 |
| *Accipiter nisus* | 63 | 19 | 62 | 18 | 0.29 |
| *Acrocephalus arundinaceus* | 2 | 0 | 2 | 0 | (0.00) |
| *Acrocephalus dumetorum* | 5 | 0 | 5 | 0 | (0.00) |
| *Acrocephalus palustris* | 14 | 0 | 14 | 0 | 0.00 |
| *Acrocephalus schoenobaenus* | 862 | 25 | 844 | 7 | 0.01 |
| *Acrocephalus scirpaceus* | 153 | 7 | 151 | 5 | 0.03 |
| *Actitis hypoleucos* | 2 | 0 | 2 | 0 | (0.00) |
| *Aegithalos caudatus* | 11 | 1 | 10 | 0 | 0.00 |
| *Aegolius funereus* | 69 | 34 | 42 | 7 | 0.17 |
| *Alauda arvensis* | 1 | 0 | 1 | 0 | (0.00) |
| *Anthus cervinus* | 7 | 1 | 7 | 1 | (0.14) |
| *Anthus pratensis* | 19 | 10 | 11 | 2 | 0.18 |
| *Anthus trivialis* | 36 | 21 | 19 | 4 | 0.21 |
| *Apus apus* | 1 | 0 | 1 | 0 | (0.00) |
| *Arenaria interpres* | 3 | 0 | 3 | 0 | (0.00) |
| *Asio flammeus* | 11 | 0 | 11 | 0 | 0.00 |
| *Asio otus* | 41 | 14 | 39 | 12 | 0.31 |
| *Bombycilla garrulus* | 2 | 0 | 2 | 0 | (0.00) |
| *Bubo bubo* | 1 | 1 | 1 | 1 | (1.00) |
| *Bucephala clangula* | 2 | 0 | 2 | 0 | (0.00) |
| *Calidris alpina* | 96 | 0 | 96 | 0 | 0.00 |
| *Calidris canutus* | 4 | 0 | 4 | 0 | (0.00) |
| *Calidris ferruginea* | 2 | 0 | 2 | 0 | (0.00) |
| *Calcarius lapponicus* | 3 | 0 | 3 | 0 | (0.00) |
| *Calidris maritima* | 1 | 0 | 1 | 0 | (0.00) |
| *Calidris temminckii* | 3 | 0 | 3 | 0 | (0.00) |
| *Caprimulgus europaeus* | 3 | 3 | 2 | 2 | (1.00) |
| *Carduelis carduelis* | 2 | 0 | 2 | 0 | (0.00) |
| *Carduelis chloris* | 556 | 83 | 531 | 58 | 0.11 |
| *Carpodacus erythrinus* | 28 | 3 | 28 | 3 | 0.11 |
| *Carduelis hornemanni* | 2 | 0 | 2 | 0 | (0.00) |
| *Carduelis flammea* | 31 | 19 | 12 | 0 | 0.00 |
| *Carduelis flavirostris* | 2 | 0 | 2 | 0 | (0.00) |
| *Carduelis spinus* | 580 | 48 | 567 | 35 | 0.06 |
| *Certhia familiaris* | 17 | 3 | 15 | 1 | 0.07 |
| *Charadrius dubius* | 6 | 0 | 6 | 0 | (0.00) |
| *Charadrius hiaticula* | 8 | 0 | 8 | 0 | (0.00) |
| *Eudromias morinellus* | 5 | 0 | 5 | 0 | (0.00) |
| *Cinclus cinclus* | 4 | 0 | 3 | 0 | (0.00) |
| *Circus aeruginosus* | 6 | 0 | 6 | 0 | (0.00) |
| *Coccothraustes coccothraustes* | 1 | 1 | 0 | 0 | - |
| *Columba palumbus* | 3 | 0 | 3 | 0 | (0.00) |
| *Corvus corax* | 2 | 0 | 2 | 0 | (0.00) |
| *Crex crex* | 1 | 0 | 1 | 0 | (0.00) |
| *Cuculus canorus* | 1 | 1 | 1 | 1 | (1.00) |
| *Delichon urbicum* | 1 | 0 | 1 | 0 | (0.00) |
| *Dendrocopos leucotos* | 2 | 1 | 2 | 1 | (0.50) |
| *Dendrocopos major* | 48 | 23 | 25 | 0 | 0.00 |
| *Dendrocopos minor* | 3 | 1 | 3 | 1 | (0.33) |
| *Dryocopus martius* | 6 | 2 | 4 | 0 | (0.00) |
| *Emberiza citrinella* | 81 | 26 | 66 | 11 | 0.17 |
| *Emberiza hortulana* | 17 | 0 | 17 | 0 | 0.00 |
| *Emberiza rustica* | 4 | 3 | 2 | 1 | (0.50) |
| *Emberiza schoeniclus* | 491 | 54 | 448 | 11 | 0.02 |
| *Erithacus rubecula* | 495 | 106 | 405 | 16 | 0.04 |
| *Falco tinnunculus* | 5 | 0 | 5 | 0 | (0.00) |
| *Ficedula hypoleuca* | 121 | 22 | 107 | 8 | 0.07 |
| *Ficedula parva* | 4 | 0 | 4 | 0 | (0.00) |
| *Fringilla coelebs* | 258 | 37 | 240 | 19 | 0.08 |
| *Fringilla montifringilla* | 71 | 14 | 63 | 6 | 0.10 |
| *Gallinago gallinago* | 10 | 3 | 4 | 0 | (0.00) |
| *Garrulus glandarius* | 13 | 4 | 9 | 0 | (0.00) |
| *Glaucidium passerinum* | 18 | 4 | 17 | 3 | 0.18 |
| *Hippolais icterina* | 25 | 0 | 25 | 0 | 0.00 |
| *Hirundo rustica* | 251 | 15 | 240 | 4 | 0.02 |
| *Jynx torquilla* | 11 | 0 | 11 | 0 | 0.00 |
| *Lanius collurio* | 9 | 0 | 9 | 0 | (0.00) |
| *Lanius excubitor* | 4 | 0 | 4 | 0 | (0.00) |
| *Larus canus* | 3 | 0 | 3 | 0 | (0.00) |
| *Limicola falcinellus* | 1 | 0 | 1 | 0 | (0.00) |
| *Locustella fluviatilis* | 4 | 2 | 2 | 0 | (0.00) |
| *Locustella luscinioides* | 4 | 0 | 4 | 0 | (0.00) |
| *Locustella naevia* | 1 | 0 | 1 | 0 | (0.00) |
| *Loxia curvirostra* | 2 | 2 | 0 | 0 | - |
| *Loxia pytyopsittacus* | 3 | 0 | 3 | 0 | (0.00) |
| *Luscinia luscinia* | 35 | 2 | 35 | 2 | 0.06 |
| *Luscinia megarhynchos* | 1 | 0 | 1 | 0 | (0.00) |
| *Luscinia svecica* | 51 | 24 | 27 | 0 | 0.00 |
| *Motacilla alba* | 25 | 20 | 8 | 3 | (0.38) |
| *Motacilla flava* | 21 | 2 | 19 | 0 | 0.00 |
| *Muscicapa striata* | 30 | 14 | 18 | 2 | 0.11 |
| *Numenius arquata* | 5 | 0 | 5 | 0 | (0.00) |
| *Panurus biarmicus* | 7 | 1 | 6 | 0 | (0.00) |
| *Parus ater* | 46 | 2 | 44 | 0 | 0.00 |
| *Parus caeruleus* | 590 | 46 | 551 | 7 | 0.01 |
| *Parus cristatus* | 20 | 5 | 16 | 1 | 0.06 |
| *Parus major* | 1280 | 187 | 1132 | 39 | 0.03 |
| *Parus montanus* | 41 | 5 | 38 | 2 | 0.05 |
| *Passer domesticus* | 12 | 6 | 6 | 0 | (0.00) |
| *Passer montanus* | 78 | 59 | 20 | 2 | 0.10 |
| *Phalaropus lobatus* | 28 | 0 | 28 | 0 | 0.00 |
| *Philomachus pugnax* | 2 | 0 | 2 | 0 | (0.00) |
| *Phoenicurus phoenicurus* | 31 | 2 | 29 | 0 | 0.00 |
| *Phylloscopus collybita* | 118 | 7 | 111 | 0 | 0.00 |
| *Phylloscopus trochiloides* | 2 | 0 | 2 | 0 | (0.00) |
| *Phylloscopus trochilus* | 524 | 71 | 474 | 21 | 0.04 |
| *Phylloscopus sibilatrix* | 14 | 2 | 12 | 0 | 0.00 |
| *Picus canus* | 2 | 1 | 2 | 1 | (0.50) |
| *Pica pica* | 3 | 3 | 2 | 2 | (1.00) |
| *Picoides tridactylus* | 4 | 0 | 4 | 0 | (0.00) |
| *Plectrophenax nivalis* | 3 | 0 | 3 | 0 | (0.00) |
| *Pluvialis apricaria* | 2 | 0 | 2 | 0 | (0.00) |
| *Pluvialis squatarola* | 1 | 0 | 1 | 0 | (0.00) |
| *Porzana porzana* | 1 | 0 | 1 | 0 | (0.00) |
| *Prunella modularis* | 420 | 34 | 393 | 9 | 0.02 |
| *Pyrrhula pyrrhula* | 218 | 37 | 216 | 35 | 0.16 |
| *Regulus regulus* | 58 | 8 | 50 | 0 | 0.00 |
| *Saxicola rubetra* | 5 | 1 | 4 | 0 | (0.00) |
| *Scolopax rusticola* | 2 | 1 | 1 | 0 | (0.00) |
| *Sterna paradisaea* | 19 | 0 | 19 | 0 | 0.00 |
| *Sterna caspia* | 4 | 0 | 4 | 0 | (0.00) |
| *Stercorarius longicaudus* | 12 | 0 | 12 | 0 | 0.00 |
| *Strix aluco* | 2 | 0 | 2 | 0 | (0.00) |
| *Strix nebulosa* | 4 | 0 | 4 | 0 | (0.00) |
| *Sturnus vulgaris* | 3 | 2 | 1 | 0 | (0.00) |
| *Surnia ulula* | 3 | 3 | 0 | 0 | - |
| *Sylvia atricapilla* | 72 | 6 | 67 | 1 | 0.01 |
| *Sylvia borin* | 84 | 6 | 79 | 1 | 0.01 |
| *Sylvia communis* | 80 | 6 | 75 | 2 | 0.03 |
| *Sylvia curruca* | 190 | 10 | 187 | 7 | 0.04 |
| *Sylvia nana* | 1 | 0 | 1 | 0 | (0.00) |
| *Sylvia nisoria* | 1 | 0 | 1 | 0 | (0.00) |
| *Tarsiger cyanurus* | 1 | 0 | 1 | 0 | (0.00) |
| *Tringa glareola* | 12 | 0 | 12 | 0 | 0.00 |
| *Tringa nebularia* | 2 | 0 | 2 | 0 | (0.00) |
| *Tringa totanus* | 1 | 0 | 1 | 0 | (0.00) |
| *Troglodytes troglodytes* | 15 | 0 | 15 | 0 | 0.00 |
| *Turdus iliacus* | 31 | 20 | 13 | 2 | 0.15 |
| *Turdus merula* | 198 | 66 | 135 | 7 | 0.05 |
| *Turdus philomelos* | 51 | 25 | 31 | 5 | 0.16 |
| *Turdus pilaris* | 37 | 26 | 15 | 4 | 0.27 |
| *Turdus viscivorus* | 2 | 1 | 1 | 0 | (0.00) |
| *Vanellus vanellus* | 21 | 0 | 21 | 0 | 0.00 |
