# Supplementary material for: Promiscuous specialists: Host specificity patterns among generalist louse flies
Source: PLoS One. 2021 May 27;16(5):e0247698. doi: 10.1371/journal.pone.0247698 (PMC8158981; doi:10.1371/journal.pone.0247698)
Supplement: S2 Table — (DOCX) [file pone.0247698.s003.docx]

**S2 Table**. Occurrences of six bird fly species (C. hir = *Crataerina hirundinis,* C. pal = *Crataerina pallida,* O. avi *= Ornithomya avicularia,* O. chl = *Ornithomya chloropus,* O. fri = *Ornithomya fringillina,* O. met = *Ornithophila metallica*) according to their observed host bird species. *Additional host records from injured birds treated in Korkeasaari Zoo, which were not included in the statistical analyses. **Observation by J. Pohjoismäki, not included in the statistical analyses.

| Species | *C. hir* | *C. pal* | *O. avi* | *O. chl* | *O. fri* | *O. met* |
| --- | --- | --- | --- | --- | --- | --- |
| *Accipiter gentilis* |  |  | X | X |  |  |
| *Accipiter nisus* |  |  | X |  |  |  |
| *Acrocephalus dumetorum* |  |  |  | X |  |  |
| *Acrocephalus schoenobaenus* |  |  |  | X |  |  |
| *Acrocephalus scirpaceus* |  |  | X |  |  |  |
| *Aegolius funereus* |  |  | X | X |  |  |
| *Anthus trivialis* |  |  | X | X |  |  |
| *Apus apus* |  | X |  |  |  |  |
| *Asio otus* |  |  | X |  |  |  |
| *Bubo bubo** |  |  | X |  |  |  |
| *Buteo buteo* |  |  |  | X |  |  |
| *Carduelis chloris* |  |  | X | X |  |  |
| *Carduelis flammea* |  |  |  | X |  |  |
| *Carduelis spinus* |  |  | X | X | X |  |
| *Certhia familiaris* |  |  |  |  | X |  |
| *Circus aeruginosus* |  |  |  | X |  |  |
| *Circus cyaneus* |  |  |  | X |  |  |
| *Columba palumbus** |  |  | X |  |  |  |
| *Corvus corone** |  |  | X |  |  |  |
| *Corvus monedula* |  |  | X |  |  |  |
| *Crex crex* |  |  |  | X |  |  |
| *Dendrocopos major* |  |  | X | X |  |  |
| *Emberiza citrinella* |  |  |  | X |  |  |
| *Emberiza rustica* |  |  |  | X |  |  |
| *Emberiza schoeniclus* |  |  | X | X | X |  |
| *Erithacus rubecula* |  |  |  | X | X |  |
| *Falco peregrinus* |  |  |  | X |  |  |
| *Falco subbuteo* |  |  | X | X |  |  |
| *Falco tinnunculus* |  |  | X | X |  |  |
| *Ficedula hypoleuca* |  |  | X |  | X |  |
| *Fringilla coelebs* |  |  | X | X |  |  |
| *Fringilla montifringilla* |  |  |  | X | X |  |
| *Gallinago media** |  |  |  | X |  |  |
| *Grus grus* |  |  | X | X |  |  |
| *Hirundo rustica* | X |  | X | X |  |  |
| *Jynx torquilla* |  |  |  | X |  |  |
| *Larus ridibundus** |  |  | X |  |  |  |
| *Luscinia svecica* |  |  |  | X |  |  |
| *Motacilla alba* |  |  | X |  |  |  |
| *Motacilla flava* |  |  |  | X |  |  |
| *Muscicapa striata* |  |  | X |  |  | X |
| *Numenius arquata* |  |  |  | X |  |  |
| *Pandion haliaetus* |  |  | X |  |  |  |
| *Parus ater* |  |  |  |  | X |  |
| *Parus caeruleus* |  |  |  |  | X |  |
| *Parus cristatus* |  |  |  |  | X |  |
| *Parus major* |  |  | X | X | X |  |
| *Parus montanus* |  |  |  |  | X |  |
| *Passer domesticus* |  |  |  | X |  |  |
| *Passer montanus* |  |  | X | X |  |  |
| *Pernis apivorus* |  |  | X | X |  |  |
| *Phylloscopus collybita* |  |  |  |  | X |  |
| *Phylloscopus trochilus* |  |  | X | X | X |  |
| *Prunella modularis* |  |  | X | X | X |  |
| *Pyrrhula pyrrhula* |  |  |  | X |  |  |
| *Regulus regulus* |  |  |  |  | X |  |
| *Saxicola rubetra* |  |  |  | X |  |  |
| *Scolopax rusticola* |  |  | X | X |  |  |
| *Sterna hirundo* |  |  |  | X |  |  |
| *Strix aluco** |  |  | X |  |  |  |
| *Sylvia borin* |  |  | X | X | X |  |
| *Sylvia communis* |  |  |  | X |  |  |
| *Sylvia curruca* |  |  | X | X |  |  |
| *Tetrao tetrix*** |  |  | X |  |  |  |
| *Turdus iliacus* |  |  | X | X |  |  |
| *Turdus merula* |  |  | X | X |  |  |
| *Turdus philomelos* |  |  | X | X | X |  |
| *Turdus pilaris* |  |  | X | X |  |  |
