# Supplementary material for: Promiscuous specialists: Host specificity patterns among generalist louse flies
Source: PLoS One. 2021 May 27;16(5):e0247698. doi: 10.1371/journal.pone.0247698 (PMC8158981; doi:10.1371/journal.pone.0247698)
Supplement: Attachment — Submitted filename: Response_to_reviewers.docx [file pone.0247698.s008.docx]

**Response to the reviewers**

**(replies in black font)**

**Reviewer #1:**

Given this report and the literature, I believe this to be an interesting manuscript and important to the readers in the field of parasitology, ecology, ornithology, wildlife and entomology. Also, the article may have an important impact and may draw attention to the problem of hippoboscids in migratory birds. English language is proper for such studies. Based on my experience statistics and molecular studies, are performed properly, and overall methodology is valid. The results are presented correctly. In particular, the discussion has been written extremely exhaustively and shows very well the important role of this research. In several points, the introduction and methodology requires reflection

A: First of all - thank you for your kind words. We are pleased to hear that that the reviewer finds louse fly ecology, a rather specialized aspect in biodiversity research, interesting.

- The end of the introduction contains a lot of information that, in my opinion, should be found in other sections of the article (e.g. results or materials and methods). Some of the methodology statements should be included in the discussion. I would very much like the authors to look at the text again and to distribute it correctly. I have marked the most important ones in the attached file.

A: We feel that the introduction needs a short explanation of the work. We hope that the reviewer is satisfied with the compromise we have tried to now make here. Similarly, L156-9: We feel that this is important information for the data included in the GLMM and therefore should be mentioned here and not in the discussion.

- All statistical abbreviations should be written in italics.

A: Corrected, thank you.

-In the attachment I am sending an article with suggestions that require reflection.

A: We have now made the suggested changes, apart for the suggestions for:

L67: Parentheses around the author names are used only when the genus name has been changed since the original description of the species. For example, Olfersia fumipennis was originally described as Lynchia fumipennis, Sahlberg but W. Dale transferred the species to the genus Olfersia in 1969 and therefore the author is written in parenthesis.

L134: We understand the confusion and now specify that Melophagus ovinus is almost extinct from Finland, but not globally.

L156-9: We feel that this is important information for the data included in the GLMM and therefore should be mentioned here and not in the discussion.

**Reviewer #2:**

The authors of the manuscript “Promiscuous specialists: Host specificity patterns among generalist louse flies” have accumulated comprehensive host association dataset of bird attacking louse flies in Finland to understand the ecological requirements of generalist species of the group. The sample sizes are very impressive and the study has adequately addressed patterns that could explain the species variation and abundances of hippoboscid flies in different host species. The authors have also provided DNA barcodes of a majority of the Finnish species of louse flies which is an important resource for future quick and precise identifications of louse flies. The study is however limited to a single country but does provide the baseline for understanding host specific patterns in an enigmatic fly group.

Here are some general comments on the manuscript are below. I’ve also included some comments/edits on the pdf.

A: Noted, thank you. We have made the suggested edits. Referring to table S3 was an accidental leftover from a previous manuscript version. Note that we have now added different tables S3 and S4, which contain the datasets used in the statistical analysis.

-The “Species determination and DNA barcoding” method section needs more details. Specifics on the primers, length of barcode used and sequencing platform used to generate the barcode sequence. Was there any post processing of raw data before the calculation of sequence divergences and NJ tree building?

A: Details of the COI sequencing added. DNA barcoding is often done in a connection of national and international barcoding initiatives, meaning that a variety of protocols have been used to generate sequences. This is true also in our case, but for those generated in the Canadian Centre for DNA Barcoding, each record’s sequence page includes a LIMS report that provides details of the protocols used. A mention of this is now added.

-Statistical analyses: What is the motivation or criteria used when deciding the parameters here (explaining abundances of bird flies in different bird species). Is this a general suite generally used for such studies? Any other factors that could not be considered due to lack of data? Also I could not find the raw data for this. This data needs to be shared as part of the journal requirements.

A: We thank the referee for this comment. We have now clarified the motivation of the used parameters in the analyses. There are in general very common species traits in avian bird analyses, which we also predicted that could have also ecological importance here.

-It would be nice to see some images of representative flies in the manuscript especially with their interesting morphology.

A: A nice idea. We have now provided some species photos in the figure 1.

-It was not clear to me why promiscuous is used in the title.

A: The title is meant to communicate a paradox: in our study we have three louse fly species with wide host range (i.e. they are promiscuous in their host use). However, when looked in detail, the species are specialized to their corresponding host niches.
